# Knockdown of *Mythimna separata chitinase* genes *via* bacterial expression and oral delivery of RNAi effectors

**DOI:** 10.1186/s12896-017-0328-7

**Published:** 2017-02-09

**Authors:** Oyunchuluun Ganbaatar, Budao Cao, Yanan Zhang, Duran Bao, Wenhua Bao, Hada Wuriyanghan

**Affiliations:** Inner Mongolia University, No.235 West College Road, 010021 Hohhot, Inner Mongolia People’s Republic of China

**Keywords:** *Chitinase*, L4440, *Mythimna separata*, Mortality, RNA interference,siRNA

## Abstract

**Background:**

RNAi (RNA interference) is a technology for silencing of target genes *via* sequence-specific manner. RNAi technology has been used for development of anti-pathogenic crops. In 2007, development of transgenic plants resistant to insect herbivore using RNAi technology was first reported, leading to a burst of efforts aimed at exploitation of RNAi mechanism and control strategy against variety of insect species based on this technique. *Mythimna separata* belongs to noctuidae family of lepidoptera and is posing threat to crops of economic importance. Recently, outbreaks of *M. separata* severely threatens corn production in Northern China, calling for new control approaches.

**Results:**

*Chitinase* genes were chosen as the target genes as they were expressed predominantly in the gut tissue and were reported to be ideal silencing targets in several insect species. Interfering sequences against the target genes were cloned into the L4440 vector to produce sequence specific dsRNAs (double-stranded RNAs). Recombinant L4440 vectors were transformed into *Escherichia coli* strain HT115 (DE3) which was defective in dsRNA degradation activity, so preserving the dsRNA from degradation by cellular machinery. The bacteria were mixed with artificial diet and were fed to *M. separata*. We showed that oral delivery of bacterially expressed dsRNA would lead to RNAi effects in the recipient insect. Quantitative real-time PCR results showed that expression level of target *MseChi1* and *MseChi2* genes in gut tissue of *M. separata* were down-regulated after oral delivery of engineered bacteria expressing the corresponding dsRNA. Sequence-specific siRNA (small interfering RNA) was detected in recipient insects, supporting the existence of siRNA-mediated silencing effects in *M. separata*. Furthermore, knockdown of *MseChi1* and *MseChi2* resulted in increased mortality and reduced body weight of the feeding larvae.

**Conclusion:**

We reported a simple and low cost experimental procedure to silence *M. separata* endogenous gene expression. Our research provides both an experimental foundation for using RNAi technology to control *M. separata* and also a useful research tool for loss-of-function study of important developmental and regulatory genes in this insect species.

**Electronic supplementary material:**

The online version of this article (doi:10.1186/s12896-017-0328-7) contains supplementary material, which is available to authorized users.

## Background

The Oriental Armyworm *Mythimna separata* Walker (Lepidoptera: Noctuidae) is one of the most important pests of cereal crops including corn, wheat and rice. In recent years, outbreaks of *M. separata* are severely threatening corn production in Northern China. To date, control strategy toward this insect pest heavily relies on the usage of chemical pesticides [1, 2]. Besides the environmental pollution and non-specific toxicity of chemical pesticide, insect resistance toward the chemicals is also becoming a serious problem in majority of the insect pests including *M. separata*. Bt (*Bacillus thuringiensis*) toxin engineered to be expressed in transgenes is an alternative for the chemicals, but it still encountered the problem of broad-spectrum toxicity and evolution of insect resistance. In fact, development of resistance against Bt toxin was also reported for *M. separata* [3, 4].

RNAi is a natural gene regulation and antiviral defense system of eukaryotic cells. RNAi has been exploited as a technology for silencing of target genes *via* sequence-specific manner [5, 6]. There are already many examples and even practical implementation of RNAi-based technologies for development of anti-pathogenic crops. RNAi-based strategies have been used for development of genetically modified plants resistant to variety of disease agents such as bacteria, nematode and virus [7–9]. In 2007, two research groups reported the development of transgenic corn and cotton resistant to insect herbivores using RNAi technology, providing a species-specific and environmentally sound anti-insect strategy [10, 11]. Since then, successful RNAi experiments have also been reported in different insect species including some lepidopteran insects [12, 13]. To screen large-scale insect genes to identify an optimal candidate target which is effective and species-specific, there is a need to develop high-throughput method for future RNAi-based control strategy in each insect species of interest.

In this study, we propose to determine the possibility of using RNAi technology to knockdown the expression of *M. separata* genes. To date, the sources of interfering RNAs (dsRNA or siRNA) commonly utilized in insect RNAi study include in vitro synthesized dsRNA, virus-expressed dsRNA or siRNA, or transgenically expressed hairpin RNA, all of which are costly synthetic molecules or are produced from time-consuming laborious procedures. To overcome the shortages of these methods, we used here an oral delivery of bacterially derived sequence specific dsRNAs to silence *M. separata* genes. We chose chitinase encoding genes as targets in this preliminary effort. Chitin (C_8_H_13_O_5_N)_n_ is a long-chain polymer of a N-acetylglucosamine linked by β-1,4-glycosidic bond. In insects, chitin is a main structural component which lines the cuticle of foregut, hindgut, trachea, and PM (peritrophic matrix) of the midgut, and is playing structural and protective roles in insect. Chitinases, also known as chitin synthases, are family 18 glycosyl hydrolases that break down glycosidic bonds in chitin and are responsible for the hydrolysis and synthesis of chitin [14–16]. *Chitinase* gene has long been studied and used as the biocontrol molecule agent toward biotic stresses including fungi and insects [17, 18]. Functions of *chitinase* genes were heavily investigated in different insect species. Several lines of evidence suggested that RNAi silencing of *chitinase* genes led to strong phenotypic effects in different insect species including *Ostrinia nubilalis* (lepidopteran), *Tribolium castaneum* (Coleopteran), *Anopheles gambiae* (Dipteran), and *Locusta migratoria* (Neopteran) [19–22]. Two chitinase homologs were identified in *M. separata*. We utilized L4440 vector to prepare dsRNAs in the *E.coli* cells for these *chitinase* genes, and directly fed them to recipient insects by mixing with artificial diet. Molecular evidence suggested that oral delivery of bacterial dsRNA caused the knockdown of target *chitinase* gene expression, as was also evidenced by the appearance of sequence-specific siRNA in the recipient insect. Mortality increase and body weight decline were also observed in recipient insects. Our data provided both theoretical and applicational direction toward the use of RNAi as a reverse genetic tool for studying the function of specific genes and also as a biocontrol method for the control of this insect species.

## Results

### Phylogenetic analysis and expression profiles of two *M. separata* genes coding for putative chitinase

**Fig. 1:**
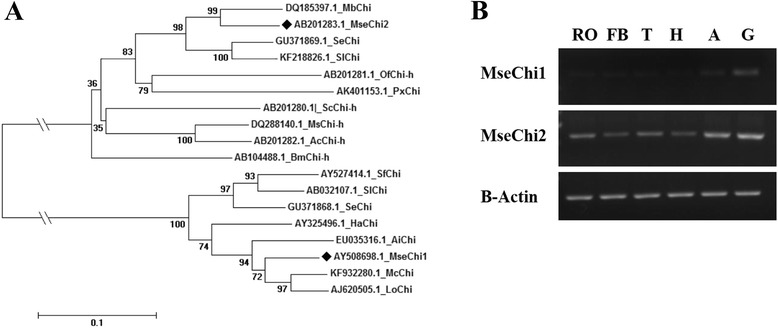
Phylogenetic analysis and expression patterns of *M. separata chitinase* genes. **a** Phylogenetic analysis of *MseChi1* and *MseChi2* by aligning with putative chitinase and chitinase-like protein encoding genes from other insect species using MEGA 6.0 software. Abbreviations, Mse: *Mythimna separata*. Mb: *Mamestra brassicae*. Se: *Spodoptera exigua*. Sl: *Spodoptera litura*. Of: *Ostrinia furnacalis*. Px: *Papilio xuthus*. Sc: *Samia cynthia*. Ms: *Manduca sexta*. Ac: *Agrius convolvuli*. Bm: *Bombyx mori*. Sf: *Spodoptera frugiperda*. Ha: *Helicoverpa armigera*. Ai: *Agrotis ipsilon*. Mc: *Mamestra configurata*. Lo: *lacanobia oleracea*. ◆ denotes *M. separata chitinase* genes. **b** Expression profiles of *MseChi1* and *MseChi2* in a variety of tissues including reproductive organ (RO), fat body (FB), thorax (T), head (H), abdomen (A) and gut (G). Semi-quantitative RT-PCR was performed for *MseChi1* and *MseChi2* using the gene specific primers at 32 cycles. 28 cycles of amplification for the house-keeping gene *β-actin* was carried out to test the integrity of tissue sample

Only 43 ESTs and 112 protein coding sequences were available for *M. separata* to date in the database of National Center for Biotechnology Information (http://www.ncbi.nlm.nih.gov). Two putative *chitinase* genes were found for *M. separata* in the database and they were named as *MseChi1* (*Mse* for *M. separata*, and *Chi* for *chitinase*) and *MseChi2* respectively. The nucleotide sequences of *MseChi1* and *MseChi2* showed high sequence similarity with other insect genes coding for chitinases. Phylogenetic analysis showed that *MseChi1* and *MseChi2* were clustered into different subfamilies (Fig. 1a). Semi-quantitative analyses of *MseChi1* and *MseChi2* transcript levels in different tissues of insect larvae were performed. As shown in Fig. 1 b, *MseChi1* and *MseChi2* were predominantly expressed in the gut tissues although their transcripts were detected also in many other tissues to a different extent. *MseChi1* and *MseChi2* appear to be ideal candidates for RNAi study *via* oral delivery of the interfering agent as the gut tissue will ingest the RNAi effectors primarily. Several lines of evidence also showed the susceptibility of gut-expressed mRNA to orally-ingested dsRNAs [12, 23–26].

### Production of dsRNAs for interfering sequences against *MseChi1* and *MseChi2*

**Fig. 2:**
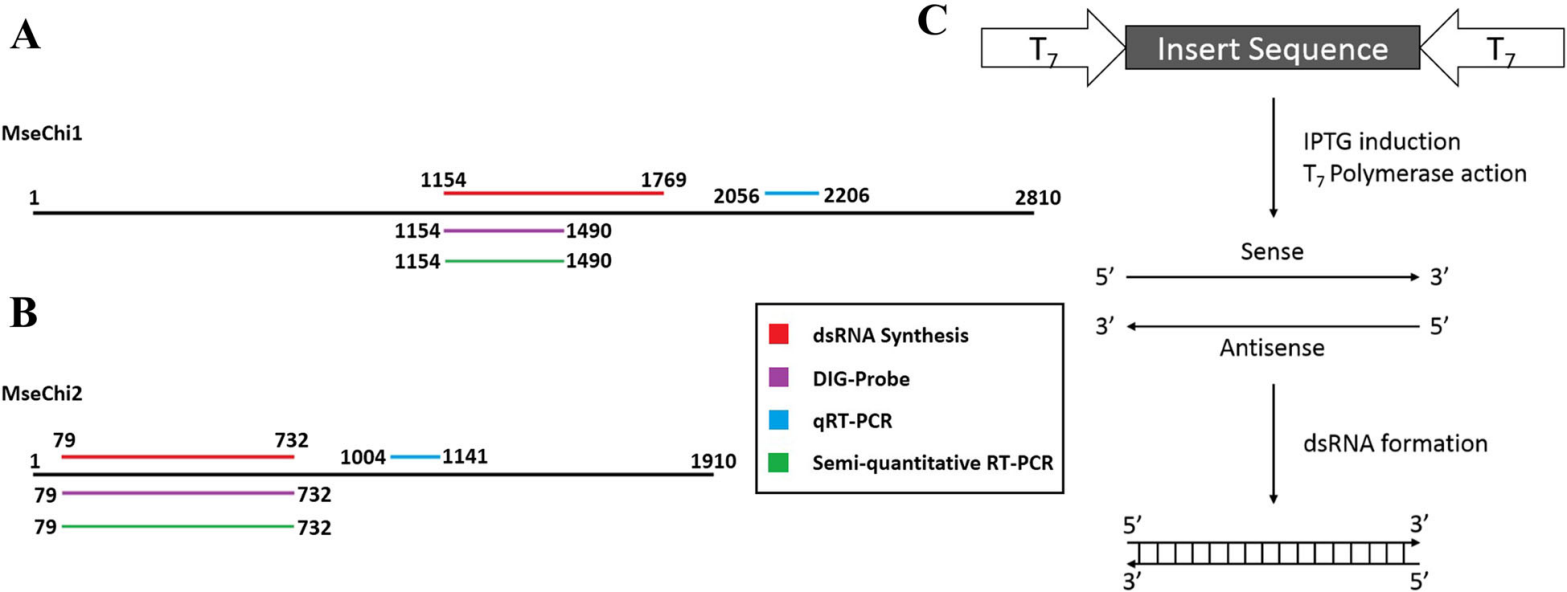
Schematics of the nucleotide fragments for different experiments. **a** Detail of the nucleotide regions used in the study for *MseChi1* gene. Red line indicates the region for dsRNA synthesis, purple line indicates the region for preparation of DIG-labelled probe, green line indicates the region for amplification of the fragment for semi-quantitative RT-PCR analysis, and blue line indicates the region for amplification of the fragments in qRT-PCR experiment. **b** Detail of the nucleotide regions used in the study for *MseChi2* gene. **c** General scheme for dsRNA production by L4440 vector in HT115 cell

**Fig. 3:**
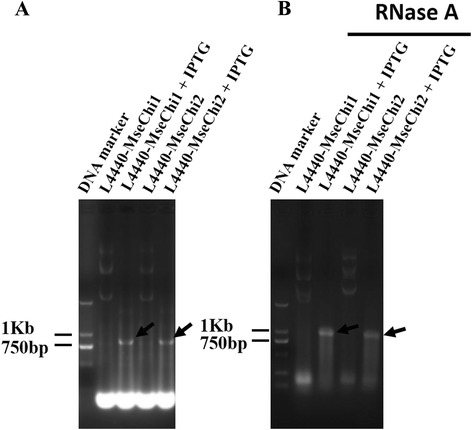
Confirmation of dsRNA produced in HT115 cell. The recombinant plasmids were transformed into HT115 competent cell. Individual transformant was cultured on 2 X YT media with or without addition of IPTG. The cell cultures were processed for total RNA extraction. RNA samples were resolved on 1% agarose gel before (**a**) or after (**b**) treatment with RNaseA. Arrowhead indicates the position of dsRNA band

**Fig. 4:**
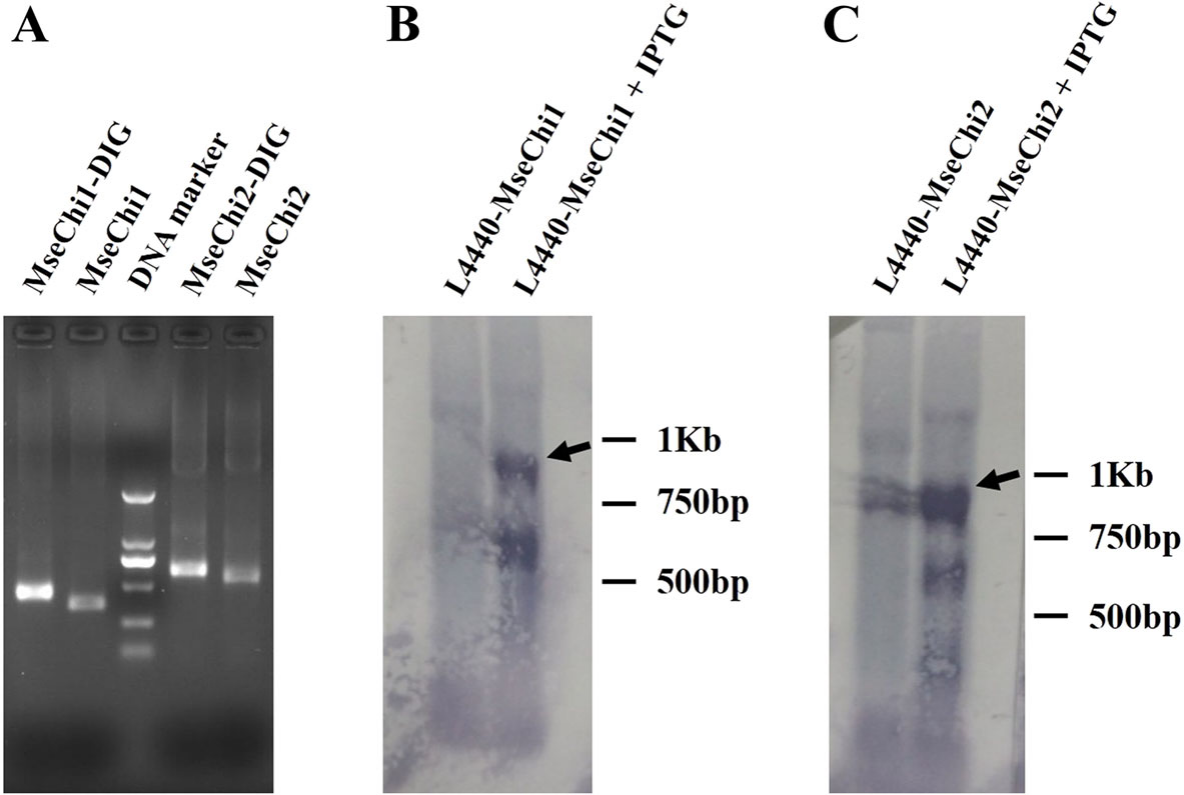
Northern hybridization detection of dsRNA produced in HT115 cells. **a** Resolution of DIG-labelled probes on agarose gel. *MseChi1* or *MseChi2* partial sequences were labelled by modified PCR with the addition of 0.05 mM of DIG-11-dUTP. DIG-labelled probes were electrophoresed on 1% agarose gel. Northern blot detection of *MseChi1* (**b**) or *MseChi2* (**c**) dsRNA after IPTG induction in HT115 cells. The cell cultures were processed for total RNA extraction. The RNA samples were resolved on 1% agarose gel after glyoxal denaturation. RNA was then transferred to nylon membranes and fixed using the crosslinker. Hybridization was carried out and the hybridized probes were finally visualized with BCIP/NBT Alkaline Phosphatase Substrate Solution

Partial fragments of *MseChi1* and *MseChi2* were amplified from the cDNA samples of *M. separata*, and the amplicons were cloned into L4440 vector. These fragments for dsRNA synthesis and those for other purposes were outlined in Fig. 2 a and b, for *MseChi1* and *MseChi2*, respectively. Two T7 promoters flanked the insert sequence in inverted orientations were expected to drive the expression of complementary RNA, resulting in the formation of dsRNA for this insert sequence (Fig. 2 c). The recombinant vectors were transformed into *E.coli* strain HT115 which lacked the double-strand-specific RNaseIII activity. T7 RNA polymerase activity was induced using IPTG (isopropyl β-D-1-thiogalactopyranoside). Total bacterial RNA was extracted, and the presence of long dsRNA segments in total RNA was analyzed. Fig. 3 a showed that *MseChi1* or *MseChi2* dsRNAs with expected size were detected only after addition of IPTG whereas they were not observed in the transformed cells without IPTG induction. The dsRNAs produced by bacteria are resistant to RNaseA treatment (Fig. 3 b), supporting their double-stranded property. It was noticed that rRNA band disappeared after IPTG induction, which was similar to the results shown before by Zhu et al. [27]. To further confirm the sequence specificity of the dsRNA band, Northern hybridization was performed using DIG (Digoxigenin)-labelled *MseChi1* or *MseChi2* sequence specific probes respectively. The probes were made by the PCR amplification of the fragments shown in Fig. 2 a, B in which DIG -dUTP was added into the reaction components. When DIG-labelled probes were separated on the agarose gel, they lagged behind the same PCR products without DIG-dUTP added. This result confirmed the efficient labelling events in that incorporation of DIG-dUTP in the probes increased their molecular mass (Fig. 4 a). As shown in Fig. 4 b and c, prevalent dsRNA bands with the expected size were detected after IPTG induction in the hybridization experiment, showing that the nucleotides corresponding to the bands are sequence specific as we expect. We also amplified the fragments from cDNAs of IPTG-induced bacteria, cloned into T-vector and verified by sequencing (data not shown).

### Confirmation of oral feeding efficiency with FD&C Blue staining

**Fig. 5:**
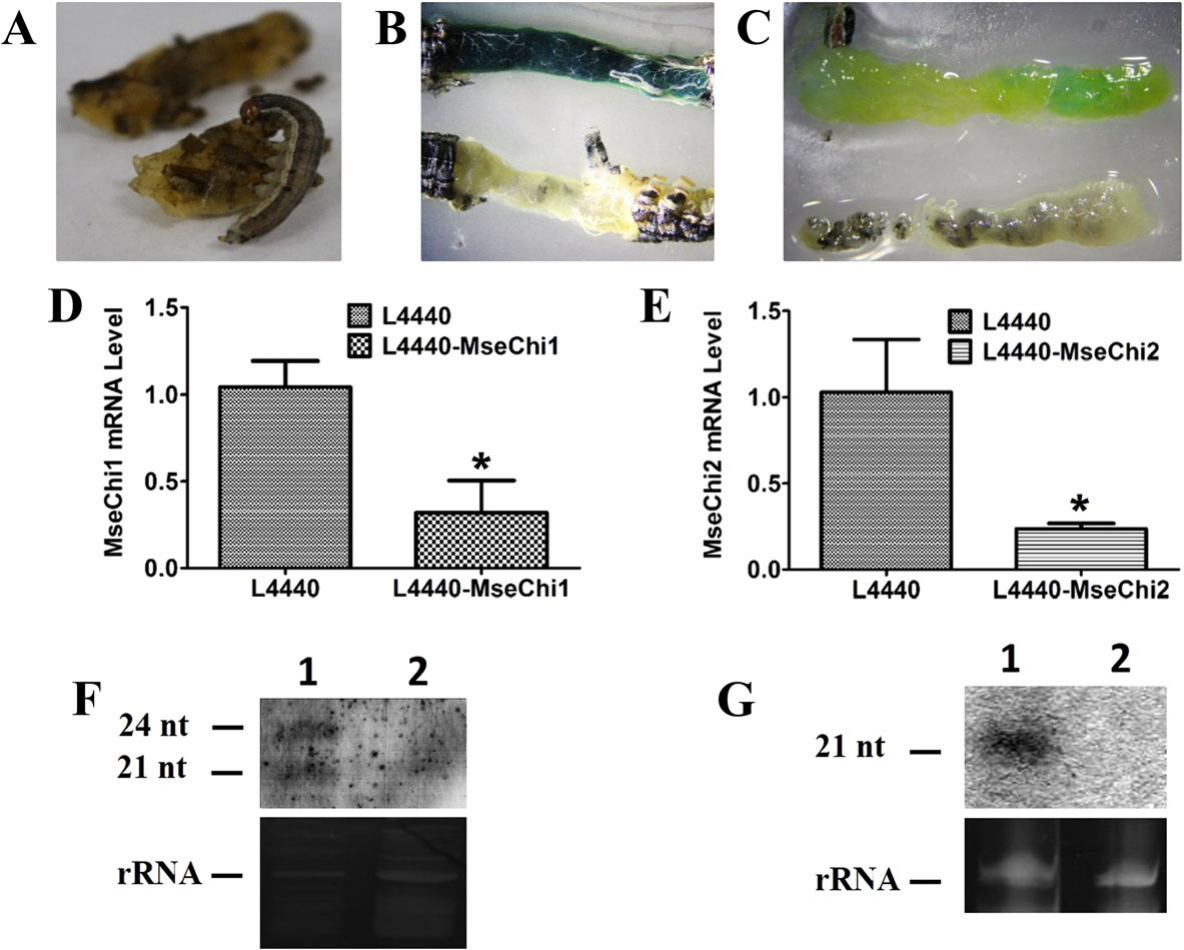
Validation for oral ingestion and molecular evidence for RNAi effects. **a** Photograph of *M. separata* larva feeding on the artificial diet. **b** Gut tissue from the larvae fed on the artificial diet mixed with (upper row) or without (lower row) FD&C Blue. **c** The same experiment with (**b**) where the intestinal content was removed from the gut tissues. **d** and (**e**) Knockdown of *M. separata chitinase* gene after feeding on the bacteria expressing dsRNAs. *T*-test was performed between treatment and control group. The single asterisk indicates a significant difference at *p* < 0.05. **f** and (**g**) Northern blot detection of small RNA in the larvae fed on dsRNA-expressing bacteria. **f** Lane 1, Larvae fed on the bacteria expressing *MseChi1* dsRNA; Lane 2, Larvae fed on the bacteria transformed with empty L4440 vector. **g** Lane 1, Larvae fed on the bacteria expressing *MseChi2* dsRNA; Lane 2, Larvae fed on the bacteria transformed with empty L4440 vector

We modified the existing formula of artificial diet for the lepidopteran insects to create a new recipe for *M. separata*. Fig. 5 a showed that *M. separata* larvae readily fed on the modified artificial diet. To verify that bacteria mixed with the artificial diet were ingested by the feeding larvae, FD&C Blue was added into the artificial diet. After six hours’ feeding, the blue diet was easily detectable inside the larvae (Fig. 5 b). When larval gut was dissected and the intestinal content was removed, the blue diet was observed in the gut tissue, demonstrating that the diet was absorbed and enriched in the gut tissue (Fig. 5 c). When the new recipe of artificial diet was compared with already reported diet formula for lepidopteran insects [28] by FD&C Blue staining, no obvious difference was observed between them (Additional file 1: Figure S1).

### Knockdown efficiency of bacterial dsRNA upon target gene expression

In order to test the silencing effects of orally-fed bacterial dsRNA on target gene expression, qRT-PCR was performed on the cDNA of gut sample for individual larva. The insect fed with bacteria carrying empty L4440 vector served as the control treatment. Expression of target genes in individual sample was normalized with the steady state level of *β-actin* gene. Insects were collected at five days after feeding, gut tissue was separated, and total RNAs were extracted. Fig. 5 d, e showed that mRNA abundance of *MseChi1* and *MseChi2* was decreased by 68% and 76% compared with the control samples, respectively. Although considerable *MseChi2* transcript was detected in non-gut tissues (Fig. 1b), RNAi effects were not observed in those tissues, demonstrating no existence of systemic movement of RNAi signal in *M. separata* (Additional file 2: Figure 2). The data strongly demonstrated that bacterial dsRNA could silence the endogenous expression of *chitinase* genes in gut tissue of *M. separata*.

### Detection of *chitinase* gene specific siRNAs

RNAi effects are not only signified by a decline in the target mRNA level, but also by the accumulation of siRNAs with sequence-specific manner. Therefore, we used Northern hybridization to test whether *chitinase* specific siRNAs were enriched in the gut tissue of bacterial dsRNA-fed larvae. Target specific siRNAs were detected only in RNAs extracted from the samples which ingested bacteria expressing dsRNA for corresponding target sequences, while the control sample showed no positive band (Fig. 5 f and g). Comparison of the positions of the siRNA band with those of RNA ladder showed that the size of siRNAs were approximately 21 nt. In the case of *MseChi1*, larger band of around 24 nt was also detected, implying that different forms of siRNA might be involved in its silencing ((Fig. 5 f). These data strongly supported the idea that knockdown of target genes in *M. separata* was mediated by the RNAi mechanism.

### Increased mortality and retarded larval growth were caused by target gene silencing

**Fig. 6:**
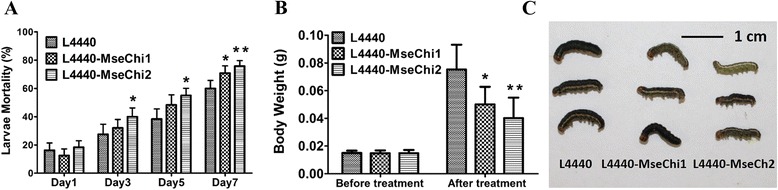
Growth changes of larvae in feeding assay. **a** Mortality rate of *M. separata* larvae. Eighty fourth instar larvae of uniform size were fed for seven days on the artificial diet containing *E.coli* cells expressing *chitinase* specific dsRNAs. Larvae fed on L4440 transformant were used as the control group. The number of surviving larvae was recorded at two days’ interval. **b** Body weights of the larvae before and after seven days’ feeding. **c** Phenotypes of larvae after seven days’ feeding. Scale bar was shown. *T*-test was performed between treatment and control group. The single asterisk indicates a significant difference at *p* < 0.05, and the double asterisks indicate a significant difference at *p* < 0.01

Phenotypic changes of recipient insects upon target *chitinase* gene silencing were investigated. Slight but significant increases in mortality rate were detected after feeding of the larvae with the bacteria expressing *chitinase* specific dsRNAs. Feeding of L4440-MseChi1 transformant caused 10.8% increase in mortality compared with the control L4440 transformant group at seven days after feeding. Feeding of L4440-MseChi2 transformant caused mortality increases from three to seven days after feeding, and the biggest mortality increase of 16.7% was observed at five days after feeding (Fig. 6 a). At seven days after feeding, body weights of surviving larvae in L4440, L4440-MseChi1 and L4440-MseChi2 groups were 0.0752 ± 0.0180 g, 0.0501 ± 0.0126 g and 0.0402 ± 0.0147 g, respectively. Therefore, body weights in L4440-MseChi1 and L4440-MseChi2 groups were decreased by 33.3% and 46.5% compared with the control L4440 group respectively (Fig. 6 b). However, no obvious deformity was observed in L4440-MseChi1 and L4440-MseChi2 groups (Fig. 6 c).

## Discussion

RNAi technique has been exploited as a temporal knockdown approach for inhibiting target gene expression. Recently, RNAi has been achieved in many insect species. *M. separata* is an economically important pest posing threat to corn production. Our aim is to examine and establish proof-of-concept for RNA interference in *M. separata*. In terms of delivery route of RNAi effector molecules toward recipient insects, there are several generally adopted ways including soaking, injection and feeding. Soaking is amenable to small animals like *C.elegans*, while it apparently seems not suitable for the insects like *M. separata* [29]. Microinjection delivers exact amounts of effector molecules into hemoceol, but it is laborious and disruptive. Feeding is a non-invasive method compared with microinjection as the former does not physically harm the insects and is also less laborious. In fact, oral feeding has been effective with the introduction of different types of RNAi effectors including in vitro synthesized dsRNA [23], siRNA [25], virus-derived RNA [30] and transgenically expressed hairpin RNA [11]. Among them, in vitro synthesized dsRNA remained as the most common form of effector molecules, while the high cost for production of it hindered its application as a widespread protocol for large-scale applications. As an alternative to in vitro synthesized dsRNA, RNAi has been shown to be achieved through voluntary ingestion of *E.coli* expressing dsRNA complementary to the target gene in *C. elegans* [31]. From then on, *E.coli* expressing dsRNAs or crude extracts of it have been used to inhibit replication of virus or silence cognate genes in insects and planarians etc. [32–37]. In one instance, dsRNA expressed in the host insect also suppressed the replication of infected virus [38]. In another instance, Chen et al. used pSilent-1 vector to express TLR7 specific dsRNA in protoplast of a funus strain IfB01 and fed them to the hemipteran sucking insect *Bemisia tabaci* to cause target gene silencing [39]. These results encourage researchers to make use of bacterially or fungal expressed RNAi effectors to screen optimal target for potential RNAi application before setting out to prepare the transgenic insect-resistant plant. Protection of plant from insect attacks by direct spray or root soaking of in-vitro synthesized dsRNAs was previously reported [40, 41]. However, technical advances on cost-efficient production of in vitro dsRNA were needed to large scale application of it. In this regard, bacteria engineered to express dsRNA might be a better alternative as it is a less expensive and more productive method [42]. Direct spray of these bacteria for field protection could be realized in different insect species as preliminary efforts are now underway [43]. Most recently, isolation and engineering of symbiont bacteria for dsRNA production and delivery were also reported to be a choice for RNAi-based insecticides [44].

In terms of target genes, dozens of genes including *V-ATPase*, *cytochrome P450* and *chitinase* genes are widely used in insect RNAi studies. As chitin is an important natural amino polysaccharide and is crucial for the growth and development of insects, chitinases/chitin synthases are viewed as the ideal targets for insect RNAi experiment. We confirmed that specific dsRNAs could be produced *via* L4440-*E.coli* expression system. We developed a *M. separata* suitable artificial diet and mixed the dsRNA expressing bacteria with this diet. Our data demonstrated that ingestion of bacterially expressed dsRNA led to sensible reduction of *MseChi1* and *MseChi2* transcript abundance and also caused accumulation of corresponding siRNA in target specific manner. Furthermore, knockdown of *MseChi1* and *MseChi2* caused slight increase in larval mortality and obvious decrease in body weight, while no visible deformities were observed. We used long dsRNA of around 700 bp in size in our experiment. Several pioneer experiments in other insect species including Western Corn Rootworm and *Tribolium castaneum*, both of which are chewing herbivorous insects, demonstrated that longer dsRNA were more readily and efficiently uptaken by the midgut tissues, leading to more pronounced RNAi effects [45, 46]. It is also an important factor to be considered that consistent supply of RNAi effectors is necessary for efficient silencing of cognate RNA in the recipient insect as the effects seem to be temporal. Although in some instance lepidopteran adult was also used for RNAi feeding experiment [23], we used only larvae to test RNAi phenomena in *M. separata* in that the larvae is most voracious in its all life stages. In our experiment, fourth instar *M. separata* larvae readily fed on the artificial diet and therefore acquired the effector RNAs in a persistent manner with high amount.

As RNAi mechanism is conserved across different organisms, RNAi effectors such as dsRNA and siRNA produced in one species could be effective toward their complementary mRNA target in other species. We detected siRNAs with the size of around 21 nt in the recipient insects, demonstrating that RNAi phenomenon is present in *M. separata*. Further analysis on RNAi-mechanism related components including AGO (Argonaute), DCL (Dicer-like) and RNA transport and amplification related proteins like SID (Systemic RNA Interference Deficiency) and RdRp (RNA dependent RNA polymerase) is needed to mechanistically support the present data. Further studies like transcriptome sequencing and screening of RNAi mechanism related proteins are underway on this aspect and we will present these data elsewhere.

## Conclusions

*M. separata* belongs to noctuidae family and is severely threatening corn production in China. Efficient genetic tools and methods are lacking in this species. We showed here that oral feeding of bacterially expressed dsRNA would cause effective RNAi knockdown effects in the recipient insect. Decreased mRNA transcript level and appearance of sequence-specific siRNA confirmed the existence of dsRNA-mediated silencing efficiency in *M. separata*. Our result presents an opportunity to make use of bacteria as the efficient producer of RNAi effectors against *M. separata*. It also provides a large-scale screening method for selection and future exploitation of ideal RNAi triggers provided we have enough candidate genes available by other methods like transcriptome sequencing. For non-model organism like *M. separata*, this method is also helpful for the study of essential gene functions by handy loss-of-function manipulation.

## Methods

### Sequence analysis of *M. separata chitinase* genes

Two putative chitinase encoding genes were found for *M. separata* in National Center for Biotechnology Information (http://www.ncbi.nlm.nih.gov/) database and they were named as *MseChi1* (Accession number AY508698.1) and *MseChi2* (Accession number AB201283.1), respectively. The software MEGA 6.0 was used for multiple alignments with putative chitinase and chitinase-like coding genes from related insect species to construct a phylogenetic tree.

### Total RNA isolation, cDNA synthesis, nucleotide sequence amplification and construction of recombinant L4440 vector

Total RNA was extracted from *M. separata* larvae using the Trizol reagents. cDNA was synthesized from one μg of total RNA using GoScript Reverse Transcription System (Promega, catalog NO. A5001). Partial interference sequence of *MseChi1* was amplified using the primer pairs of 5’-GCGGCCGCgggactttgtggacctgaga-3’ and 5’-GCGGCCGCctgtcttgctcggcgaatac-3’; Partial interference sequence of *MseChi2* was amplified using the primer pairs of 5’-GCGGCCGCgtccgatgttggcgttagtc-3’ and 5’-GCGGCCGCctttgaagtcttctcggccg-3’, where the *Not*I site is underlined. The amplicons were digested with *Not*I and ligated into the *Not*I-digested L4440 vector, respectively. The recombinant vectors were validated by PCR amplification, *Not*I digestion and direct sequencing.

### Gene expression profiles in different tissues

To analyze the expression patterns of *MseChi1* and *MseChi2* in different tissues, fourth instar larvae were immobilized on an ice box for 10 min, and were dissected with forceps while examining with a dissecting microscope. Variety of tissues were collected, RNAs were isolated, and cDNAs were prepared. Partial transcript fragments of *MseChi1* and *MseChi2* described in Fig. 2 were PCR amplified. The abundance of *β-actin* (Accession number GQ856238) transcript amplified with the primers 5’-ccacgagaccacctacaact-3’ and 5’-ggaatcgacaatgttccgca-3’ was used as the internal control. For *MseChi1* and *MseChi2*, 32 cycles of amplifications were performed, and for *β-actin*, 28 cycles of PCR were performed.

### Expression and detection of dsRNA in *E.coli*

RNaseIII-deficient *E.coli* strain HT115 was grown in 2 X YT broth medium with ampicillin (100 μg/mL) and tetracycline (10 μg/mL). Recombinant L4440 vectors containing insert interference sequences were transformed into HT115 competent cells. Single colony of HT115 transformant was cultured in 2 X YT medium with the above antibiotics overnight. The bacteria culture was diluted in 2 X YT medium with the ratio of 1:100 and was allowed to grow to OD_600_ = 0.5. IPTG was added to the medium with the final concentration of 1 mM, and the bacteria were cultured for additional five hours. The bacteria were then killed by heating at 80 °C for 20 min, collected by centrifugation at 3000 rpm, and were used for total RNA isolation or larval feeding experiment described below. In order to detect the dsRNA produced in HT115 after IPTG induction, total RNA was isolated and ten μg of RNA was dissolved on 1% agarose gel in TBE buffer. Total RNA isolated from the same cultures without IPTG addition was used as control. Total RNA was treated with 0.2 μg/mL RNase to remove single-stranded RNA in some cases before electrophoresis. Gel was stained with Goldview I nucleic acid dye and was visualized under GelDoc-It^2^ Imager. DM2000 DNA ladder was run on the same gel to estimate the size of dsRNA band.

### Northern blot experiment to verify sequence specificity of dsRNA band

Ten μg of total RNAs from above experiment were denatured using glyoxal and resolved on 1% agarose gel in MOPS buffer. RNAs were then transferred to nylon membranes (Roche, catalog NO. 11209299001) in 20 X SSC buffer through the capillary blotting system. Transferred RNA on the membrane was fixed using the UV crosslinker (UVP, CL-1000). Hybridization was carried out using *MseChi1* or *MseChi2* specific probes in ULTRAhyb Buffer (Ambion, catalog NO. AM8670). *MseChi1* or *MseChi2* partial sequences were labelled with DIG-dUTP using modified PCR with 0.25 mM each of dATP, dTTP, dGTP, dCTP and 0.05 mM of DIG-11-dUTP (Roche, catalog NO. 11558706910). For *MseChi1* probe, primer sets 5’-GCGGCCGCgggactttgtggacctgaga-3’ and 5’- ggcacatcatcaacaggctt-3’ were used. For *MseChi2* probe, primer sets 5’-GCGGCCGCgtccgatgttggcgttagtc-3’ and 5’-GCGGCCGCctttgaagtcttctcggccg-3’ were used. The labelled probes were resolved on 1% agarose gel for verification. Hybridization was performed at 65 °C overnight. Blots were washed and immunodetected with the Anti-DIG-AP Fab fragments (Roche, catalog NO.11093274910) using the DIG Wash and Block Buffer Set (Roche, catalog NO. 11585762001). Hybridization signals on the membrane were finally visualized with BCIP/NBT Alkaline Phosphatase Substrate Solution and were photographed.

### Insect culture and bioassay

Laboratory-adapted *M. separata* was obtained from Institute of Plant Protection (IPP), Chinese Academy of Agricultural Sciences (CAAS). The strain had been reared on fresh corn leaves in growth chamber with the condition of 25 °C, 70% relative humidity, and a photoperiod of 14 h:10 h (light:dark). For the bacteria feeding experiments, artificial diet with the formula described in Table 1 was used. To investigate the feeding efficiency, artificial diet containing F&D Blue was used. After six hours’ feeding, the insects were dissected, gut tissues were collected, intestinal content was removed and visualized under Leica EZ4 light microscope. Newly molted fourth instar larvae of uniform size and weight were starved for 24 h and then were fed with artificial diet containing bacteria pellet. Total RNA was extracted from gut tissue of five day-fed insects and was used for the following experiments. Mortality rate of feeding larvae were recorded at two days’ interval and body weights of surviving larvae were measured at seven days after feeding.

**Table 1:** Recipe for the modified artificial diet for *M. separata*

Component A		Component B	
Corn flour	2–3 g	Glucose	1.8–2.4 g
Corn leaf flour	4–5 g	L-Ascorbic acid	0.3–0.4 g
Soybean flour	2.3–2.8 g	Distilled water	25 ml
Yeast extract	4–5 g	Component C	
Casein	1–1.5 g	Distilled water	75 ml
Cholesterol	0.06–0.08 g	Agar	1.7–2 g
		Sorbic acid	0.1–0.2 g

Leaves of corn seedling were dehydrated with oven and were ground into powder using the pestle. Component A and B were mixed, then the component C was added

### Quantitative real time PCR experiment

The effects of bacterially expressed dsRNA on target *chitinase* mRNA levels were evaluated by quantitative real-time PCR using Fast SYBR Green Master Mix (Applied Biosystems). In order to get rid of undesirable amplification from input recombinant plasmids and/or dsRNAs, primers for qRT-PCR were designed to detect target mRNAs by amplifying sequences that lay outside of the insert interfering sequences. A pair of primers 5’-tggctgctcggtgacttgat-3’ and 5’-tgttgcggcttcattgaaca-3’ were used for *MseChi1* mRNA level detection. A pair of primers 5’-cctggtggtaagggagccaa-3’ and 5’-ttccttccggtcttggcaga-3’ were used for *MseChi2* mRNA level detection. Transcript abundance of *M. separata β-actin* was used as an internal control for normalization using the primer sets 5’-cgattccgttgccctgagg-3’ and 5’-catgatcgagttgtaggtggtct-3’. Larvae fed with Bacteria transformed with L4440 vector were used as control treatment. Gene expression data were analyzed using the relative 2^–ΔΔCT^ method [47]. Three biological replications, each with two technical replications were carried out for the qRT-PCR experiment. Statistical analyses were performed between treatment and control groups with *t*-test.

### SiRNA detection

Northern hybridization was performed to investigate whether sequence specific siRNAs were accumulated in *M. separata* following feeding on bacterially expressed dsRNA. Total RNA was extracted from the gut tissue of *M. separata* after five days’ feeding and the small RNA was isolated from the total RNA using mirVana PARIS small RNA isolation kit (Ambion, catalog NO. AM1556) according to the Manufacturer’s protocol. Five μg small RNAs were separated on 15% denaturing polyacrylamide gel containing 8 M urea, and the 5S rRNA band was visualized by ethidium bromide staining. RNAs were transferred to nylon membranes using the Trans-Blot SD Semi-Dry Transfer Cell (Biorad) and fixed with 1-ethyl-3-(3-dimethylaminopropyl) carbodiimide following the protocol described in the literature [48]. DIG-labelled probes and hybridization procedure were the same to those in Northern blot experiment for dsRNA detection. MicroRNA Marker (New England Biolabs, catalog NO. N2102S) was run on the same gel to estimate the sizes of small RNAs.

## Additional files

Additional file 1: Figure S1. Photograph of *M. separata* larvae feeding on the artificial diet with different recipes. Gut tissues from the larvae fed on the FD&C blue containing artificial diet of old recipe (upper row) and new recipe developed by us (lower row) were shown. (JPG 60 kb)
Additional file 2: Figure S2. Expression analysis of *MseChi2* in non-gut tissues. *MseChi2* expression was analyzed in non-gut tissues after five days’ feeding of HT115 cell expressing *MseChi2* specific dsRNA. Larvae fed with L4440 transformant were used as control. RO, reproductive organ; FB, fat body; T, thorax; H, head; A, abdomen. (JPG 97 kb)

## Abbreviations

AGO: Argonaute
Bt: *Bacillus thuringiensis*
Chi: Chitinase
DCL: Dicer-like
DIG: Digoxigenin
dsRNA: Double-stranded RNA
IPTG: Isopropyl β-D-1-thiogalactopyranoside
MOPS: 3-(N-morpholino) propanesulfonic acid
Mse: *Mythimna separata*
PCR: Polymerase chain reaction
PM: Peritrophic matrix
PTGS: Post-transcriptional gene silencing
qRT-PCR: Quantitative real time PCR
RdRp: RNA dependent RNA polymerase
RNAi: RNA interference
SID: Systemic RNA Interference Deficiency
siRNA: Small interference RNA
