# Supplementary figures and images for: Knockdown of Mythimna separata chitinase genes via bacterial expression and oral delivery of RNAi effectors
Source: BMC Biotechnol. 2017 Feb 9;17:9. doi: 10.1186/s12896-017-0328-7 (PMC5301351; doi:10.1186/s12896-017-0328-7)

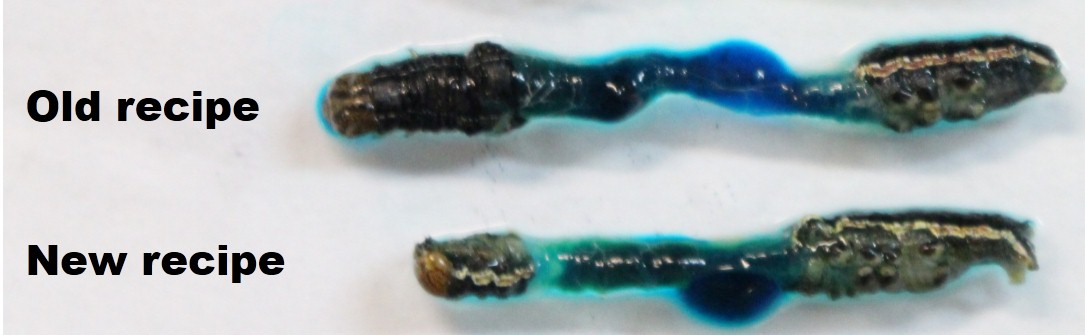

Supplement: Additional file 1: Figure S1. — Photograph of M. separata larvae feeding on the artificial diet with different recipes. Gut tissues from the larvae fed on the FD&C blue containing artificial diet of old recipe (upper row) and new recipe developed by us (lower row) were shown. (JPG 60 kb) [file 12896_2017_328_MOESM1_ESM.jpg]

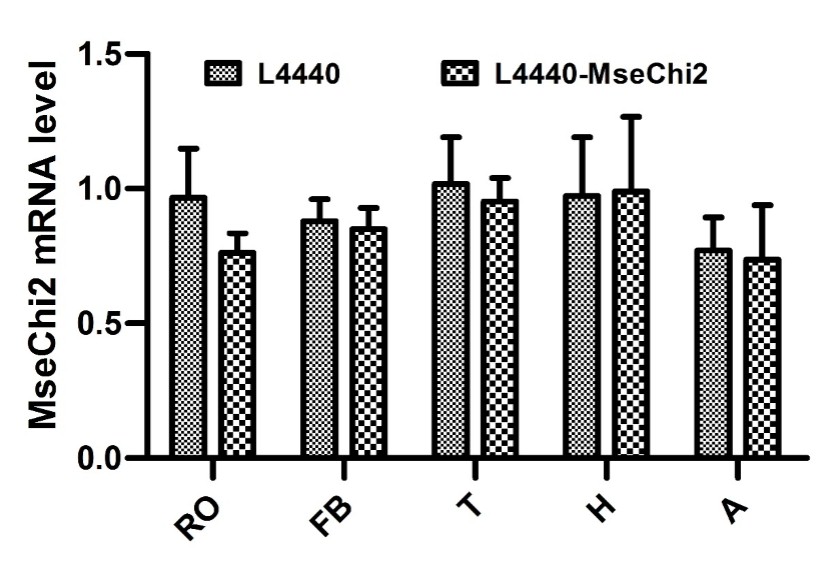

Supplement: Additional file 2: Figure S2. — Expression analysis of MseChi2 in non-gut tissues. MseChi2 expression was analyzed in non-gut tissues after five days’ feeding of HT115 cell expressing MseChi2 specific dsRNA. Larvae fed with L4440 transformant were used as control. RO, reproductive organ; FB, fat body; T, thorax; H, head; A, abdomen. (JPG 97 kb) [file 12896_2017_328_MOESM2_ESM.jpg]
